# Giant Benign Mediastinal Masses Extending into the Pleural Cavity

**DOI:** 10.1055/s-0036-1584519

**Published:** 2016-06-10

**Authors:** Güven Sadi Sunam, Murat Öncel, Sami Ceran, Kemal Ödev, Hüseyin Yıldıran

**Affiliations:** 1 Department of Thoracic Surgery, Medical Faculty, Selçuk University, Konya, Turkey; 2 Department of Thoracic Surgery, Medical Faculty, Necmettin Erbakan University, Konya, Turkey; 3 Department of Radiodiagnostics, Medical Faculty, Necmettin Erbakan University, Konya, Turkey

**Keywords:** mediastinum, huge mass, benign

## Abstract

**Introduction** The aim of the study was to evaluate the results of surgery to remove huge mediastinal masses and their pathology. Surgical resection was chosen for accurate diagnosis and treatment of the huge mediastinal masses extending into the pleural cavity.

**Methods** Records were reviewed for eight patients who had the diagnosis of huge benign mediastinal masses and who underwent operation; details of the patients and operations were recorded.

**Results** Mean age was 34.5 (range 22 to 44) years, and male-to-female ratio was 2:6. Computed tomography and magnetic resonance imaging (MRI) were used to evaluate the location and extent of the abnormality and to characterize the tissue components of the mass. Most of the tumors were located in the posterior mediastinum. The most frequent presenting symptom was exertional dyspnea. The majority of cases underwent posterolateral thoracotomy, and complete resection was possible in seven patients. Partial resection could only be performed in one. The mean diameter of the resected masses was 15 × 10 cm. Histopathologic examination revealed 3 neurogenic tumors, 2 teratomas, 1 thymolipoma, and 1 ectopic thyroid, and 1 hemangioma. Minor complication was seen in two cases.

**Conclusion** The presurgical thoracic MRI provided correct diagnosis along with radiologic characterization and topography. Surgery must be the preferred treatment in huge benign mediastinal masses.

Most mediastinal masses are primary and asymptomatic. They are diagnosed either accidentally or when they press on the adjacent anatomic tissues. Therefore, the size of the lesion is important because it plays a role in the appearance of symptoms. 1 2 Although the development of radiologic and noninvasive methods increased the rates of diagnosis before surgery, for definite diagnosis and treatment, surgery becomes inevitable. In this retrospective study, our purpose was to discuss the diagnosis and treatment of huge benign mediastinal masses extending into the pleural cavity and to review the literature.

## Methods

Eight cases with rarely seen huge mediastinal mass underwent surgical treatment in our clinic. Mediastinal cysts were not included in the study. The cases were evaluated in terms of age, sex, primary diseases, symptoms, mass size, computed tomography (CT) and magnetic resonance imaging (MRI) findings, type of surgical treatment, and histopathologic results. Full blood count, biochemical examinations, bilateral pulmonary function test, and CT were obtained in all cases and in some cases respiratory functional tests, MRI, and echocardiography were obtained. The surgical treatments applied were either median sternotomy or right/left thoracotomy. The complications during the operation and follow-up were recorded ( Table 1 ).

**Table 1:** General features of the cases

Case	Age	Sex	Complaint	Location	Surgery	Pathology
1	44	M	Hemoptysis, orthopnea	Mid	Thoracotomy	Ectopic thyroid
2	31	F	Orthopnea	Whole	Sternotomy	Thymolipoma
3	22	F	Orthopnea	Posterior	Thoracotomy	Mature teratoma
4	35	F	Orthopnea	Anterior-upper	Thoracotomy	Schwannoma
5	37	F	No	Whole	Thoracotomy	Hemangioma
6	30	F	Lumbago	Posterior	Thoracotomy	Ganglioneuroma
7	37	F	Tachycardia	Anterior	Thoracotomy	Mature teratoma
8	34	M	Orthopnea	Posterior	Thoracotomy	Neurofibroma

## Results

Two of the eight patients diagnosed and treated for huge benign tumor were men and six were women. The mean age was 33 (range 22 to 44). The general features of the patients are given in Table 1 .

Cases 1, 2, 3, 4, and 8 had respiratory problems and cases 6 and 7 had nonspecific complaints; one case presented to our clinic with trauma.

**Fig. 1:**
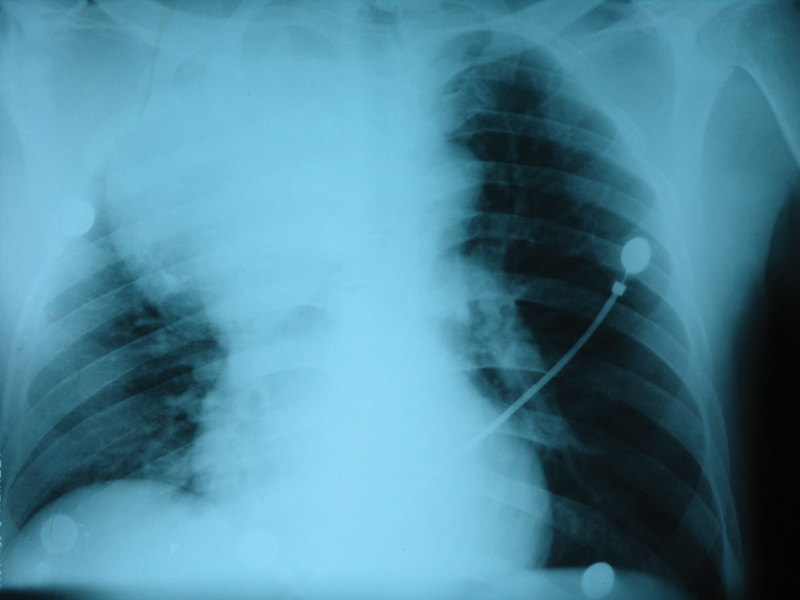
Hematoma in thorax.

The ecchymosis in case 1 was found especially in the head, thorax, and right hemithorax interior upper side, and a tubercle was seen on the right arm. The right radial pulse could not be taken. Additionally, there was crepitation and pain in the right clavicle area. Respiratory sounds could not be heard in the anterior upper right. There was bleeding from time to time as evidenced by hemoptysis of 50 to 100 mL, diagnosed as maxillofacial injury by the otorhinolaryngology department; however, it was not certain whether the bleeding was from the maxillofacial injury or from the lungs. Tachycardia and blood pressure were not stable. Due to the maxillofacial injury, bronchoscopy was not used; the patient was taken to the operation with the diagnosis of hematoma in the thorax ( Fig. 1 ).

A respiratory problem was clear in case 2; the patient could not lie back and the respiratory sounds in both lungs were decreased. In blood gas examination, hypoxia (PaO _2_ : 48 mm Hg) was found. After the tests, the patient was taken to the operating room under elective conditions with mass diagnosis.

**Fig. 2:**
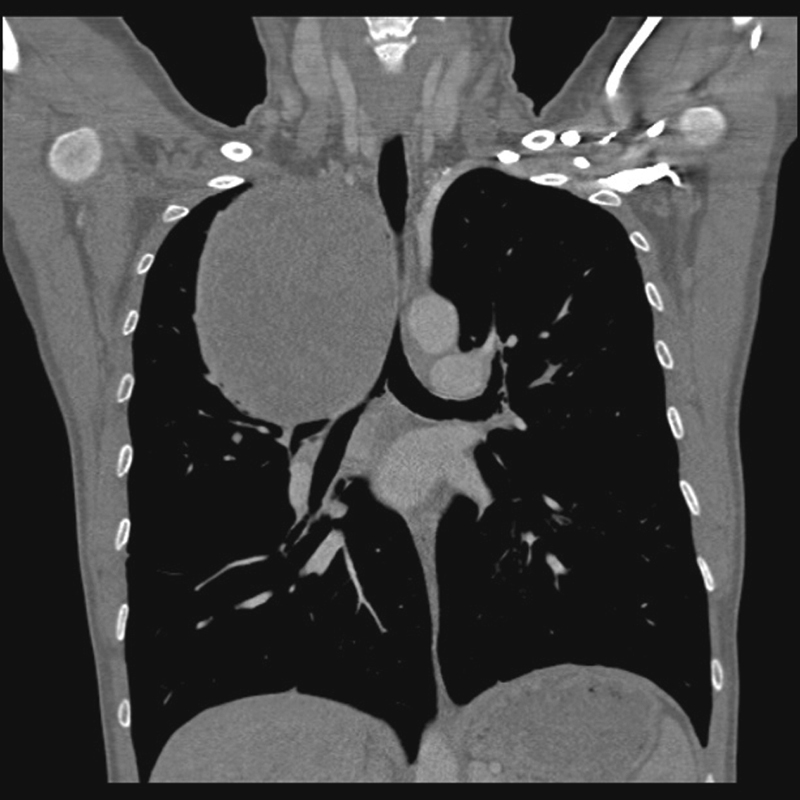
Mass located in upper mediastinum.

**Fig. 3:**
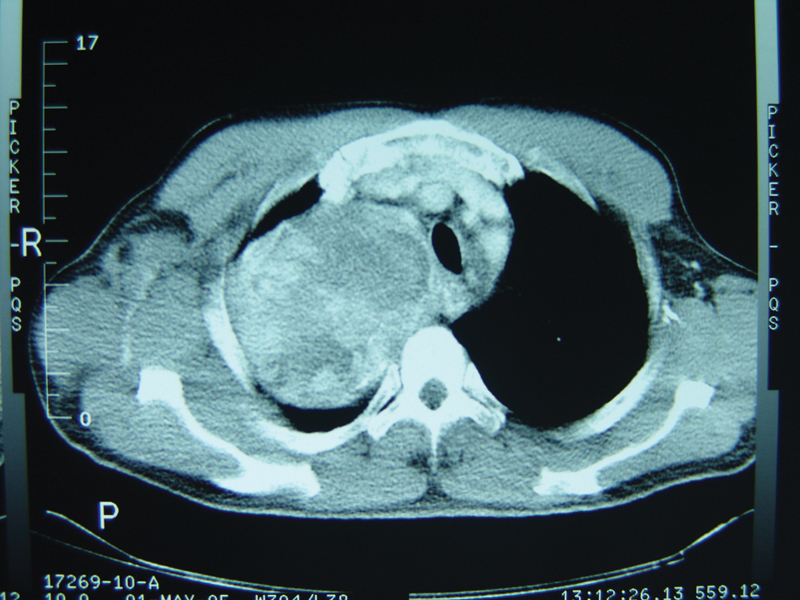
Computed tomography of giant tumor.

There was no clear examination findings but respiratory problems were evident in cases 3 and 4 ( Figs. 2 , 3 ). In the third case, the patient was examined by chest physicians, and thoracentesis was applied for pleurisy but it caused pneumothorax, because the doctor asked for echocardiography (ECHO) instead of CT. ECHO showed that the mass was not cardiomegaly, and the patient was transferred to the radiodiagnostic department for diagnosis.

In cases 5, 6, and 8, the complaints were not related to respiratory problems because there were no symptoms. There was palpation in case 7. Except case 1, all the patients had respiratory tests. Slight restrictive respiratory patterns were found in all cases except two that had severe symptoms.

Posteroanterior chest X-ray and pulmonary function tests were done for all patients. In addition to the mediastinal mass, there was a broken clavicle in case 1, pneumothorax in case 3, and pleurisy in case 8. The clavicle broke in case 1 due to trauma, the pneumothorax in case 3 was due to thoracentesis, and the pleurisy in case 8 was reactional. Thorax CT was taken for all the cases. MRI was not obtained for case 1 because it required urgent action ( Table 2 ). According to CT and MRI results, benign mediastinal mass was diagnosed for all cases except case 1. Case 1 was considered a hematoma inside the thorax. As an additional test, transthoracic fine-needle aspiration biopsy in case 8 showed mature cystic hematoma. This diagnosis differed from the postoperative pathologic examination result of neurofibroma. Although three cases originated from the posterior mediastinum, two cases originated from the anterior, one case from middle, and one case from whole mediastinum. Five cases had the right pleural cavity affected, two had extensions into the left pleural cavity, and in one case both cavities were affected.

**Table 2:** Radiologic features of the patients

Case	CT findings	MRI findings
1	12 × 7-cm lesion with no bleeding, mass distinction, on the right in upper-middle mediastinum defined, limited	No
2	Huge mass filling all mediastinum and exerting pressure on the lungs	The mass was richer than fat tissue with no neighboring tissue invasion; prediagnosis: lipoma
3	17 × 14-cm flat, bordering lobule, nonenhancing mass in left hemithorax originating from posterior mediastinum and pneumothorax	Cystic mass richer than the liquid that contains septation; prediagnosis: mature teratoma
4	12 × 10-cm solid mass exerting pressure on main bronchus and trachea in upper right mediastinum	Neurogenic tumor in upper right mediastinum
5	Nonenhancing mass extending from right paracardiac area to all mediastinum	A nodular contoured mass located and completing the whole mediastinum; prediagnosis: hemangioma
6	12 × 6-cm mass with enhancing heterogenic structure located in right paravertebral extending between T8 and L2	Heterogenic neurogenic mass, no relation with spinal canal
7	11 × 8-cm paracardiac mass on left anterior mediastinum	11 × 8-cm mature hematoma filled with liquid located on left anterior mediastinum
8	13 × 6-cm paravertebral smooth-edged mass and liquid-containing necrosis and cystic areas	13 × 6-cm solid neurogenic tumor within smooth borders and lobular contour, with no spinal connection, on the left in the pleural liquid located in right posterior mediastinum

Abbreviations: CT, computed tomography; MRI, magnetic resonance imaging.

All the cases except case 2 underwent thoracotomy. Because the mass filled the whole mediastinum in case 2, a median sternotomy was preferred. Capsuled mass excision was done in all cases except case 5. Because the mass did not leave the mediastinum completely (pathology: hemangioma), only a biopsy was taken. Anatomic pulmonary resection was not needed for any cases. In cases 3 and 7, the phrenic nerve was excised too because the mass captured the phrenic nerve on the same side. Diaphragmatic elevation developed in both cases, whereas dysphonia was only seen in case 7. No other complication was encountered. There was no recurrence during the long-term follow-up.

## Discussion

Because most of the masses originating in the lungs are small outer cell lung cancers, their diagnosis and treatment modalities are relatively uniform, and speak a common language. However, it is more difficult speaking a common language for mediastinum masses. Although the mediastinum is an anatomic area, it contains different tissues and pluripotent cells. For that reason, mediastinum masses can be benign or malignant, and malignant mediastinum tumors can be primary or secondary tumors. Primary malignant tumors are rarely seen; secondary tumors are accepted as metastatic tumors and generally result from lymphatic spread of neoplasms to the mediastinal lymph nodes. Primary mediastinum benign tumors, on the other hand, are a heterogenous group appearing in neoplastic, congenital, and inflammatory cases; they are generally accepted as asymptomatic and can reach large sizes. In a series of 2,431 cases gathered from the literature, the incidences of tumors in the mediastinum were as follows: neurogenic tumors, 21%; thymoma, 19%; lymphoma, 13%; neoplasia with germ cells, 10%; primary carcinoma, 5%; mesenchymal tumor, 6%; endocrine tumor, 6%; cysts, 18%; and others, 3%. 3 4 When the localizations are considered, the tumors seen in the anterior mediastinum generally originate in the thymus (thymoma, thymolipoma, thymic, cysts, thymus hyperplasia; 30%) or are lymphoma (20%) and germ cell tumors (18%). In 9.5 to 20% of cases, the remaining lesions are located in the middle mediastinum, and 26 to 33.4% are located in the posterior mediastinum. 4 5 6 Davis et al stated that in the middle mediastinum, the most frequent benign cystic lesions (60%) were lymphomas (21%) and benign mesenchymal tumors (9%). Of the tumors located in the posterior mediastinum, 53% were neurogenic tumors, 34% were bronchogenic and neuroenteric cysts, and some of the mediastinal masses consisted of mesenchymal tumors. 3 4 5

It has been stated that 56 to 62% of the cases in the research were symptomatic; 16% had dyspnea, 30 to 43% had chest pain, and 16% had coughing. 3 4 7 8 The symptoms mostly appear as pressure symptoms depending on the location and type of the lesion. The pressure can result from loss of volume in lungs from the tumors extending into the pleural cavity or trachea or large bronchus. We found 70% of our cases were symptomatic. We found that larger masses tended to be symptomatic. The third case was diagnosed as mature teratoma 17 × 14 cm in size and weighing 940 g in the posterior mediastinum, which is the second largest case in the literature. This mass pressed the left lung and although the patient was young, he had dyspnea. The case with thymolipoma had the noisiest symptomatology as the mass covered the whole mediastinum and invaded both pleural cavities. Case 4 had dyspnea due to trachea pressure. The location of the masses and their large size and extension into the pleural cavity increased the symptoms. The literature noted that some mature teratomas cause perforated massive pleurisy and cardiac tamponade. 9

As with other thoracic cysts, the purpose of radiologic examination and histopathologic typing in mediastinal masses is to obtain a diagnosis that will differentiate mediastinum mass from the masses that have the same prior radiologic image results so that the optimal treatment method can be planned. At the same time, it is necessary to determine compression and invasion into the tracheobronchial tree, pulmonary vascular structures, and superior vena cava and other mediastinal problems to determine the systemic problems to be encountered pre- and postoperatively. For that reason, CT provides vital information on the localization of the lesion in the mediastinum and the density of the mass, its size, and its relation with mediastinal anatomic structures, along with information on whether the mass includes liquid or calcifications and its weight and shape. However, Rendina et al stated that the rate of correct histopathologic diagnosis prediction was not very high with CT (around 68%). 10 If mediastinum pathology is to be examined with conventional or spiral CT, enhancing material should certainly be used, which can help clarify the relation between lungs, mediastinal structures, and the mass and the vascularization features of the mass. Although CT findings diagnosed the mass in all of our cases, they had limited contributions to etiology. In case 1, CT even led to diagnosis error. However, MRI with soft tissue contrast resolution has some advantages. These problem-solving methods demonstrate the relation between vascular-originating mediastinum pathologies, neurologic tumors, cystic lesions with dense content, and the relation of these lesions with pericardium, heart, and spinal canal. It enables a better and clear evaluation of the findings. With this feature, MRI seems to be a better evaluation method for mediastinal masses when compared with CT. 4 Except one case, all our cases underwent MRI examination. The findings of MRI in our cases closely complied with the results of histopathologic diagnosis from the surgically removed mass. For that reason, thoracic MRI in mediastinal masses should certainly be done and the treatment should be decided accordingly.

The evaluation of the operability of mediastinal masses is very different than that of lung cancer. The appropriate operative procedure and surgical timing depend on the patient's age, the size and type of the tumor, its localization and expansion, accompanying diseases, cardiorespiratory reserve, and the experience of the surgeon.

The type of incision used for resection is usually posterolateral thoracotomy and second median sternotomy. 11 12 However, in invasive masses or anterior mediastinum masses, median sternotomy can be preferable. 11 For this reason, in case 2 median sternotomy was used. In fact, posterolateral thoracotomy should be preferred for middle and posterior mediastinum masses. If there is medulla spinalis connection in neurogenic tumors, first the connection between the tumor and medulla spinalis should be removed with a posterior approach by the neurosurgery department and then the mass should be removed by thoracotomy. Although there was neurogenic tumor in three of our cases, no medulla spinalis connection was observed. Although video-assisted thoracoscopic surgery is preferred for smaller mediastinum masses and cysts, it is controversial in huge masses. Surgical morbidity and mortality of mediastinum masses, except rare cases, are under 10 and 1%, respectively. 13 14 The diaphragmatic elevation seen in the postoperative period can be maintained for 2 years. 4 Surgery provides complete healing in almost all of the benign cases. 13 14 15 In our second case, the phrenic nerve was injured because the mass reached a huge size that led to extraction of the phrenic nerve with the tumor.

## Conclusion

Though huge masses in the mediastinum and extending to the pleural cavity are rare, surgery should be performed after a thorough radiologic examination. Thoracic MRI is necessary to obtain a histopathologic diagnosis and eliminate the disease and the symptoms.
